# Teaching the social determinants of health through medical legal partnerships: a systematic review

**DOI:** 10.1186/s12909-021-02729-1

**Published:** 2021-05-26

**Authors:** Kristian Welch, Benjamin Robinson, Michaela Lieberman Martin, Amy Salerno, Drew Harris

**Affiliations:** 1 The University of Virginia School of Medicine, VA Charlottesville, USA; 2 The Legal Aid Justice Center and the UVA Health System, Charlottesville, USA; 3 Department of Medicine, University of Virginia, Charlottesville, VA USA; 4 Department of Medicine, Division of Pulmonary & Critical Care, University of Virginia, Charlottesville, VA USA

## Abstract

**Background:**

Undergraduate and graduate medical education often includes the social determinants of health, but questions remain regarding how best to ensure that trainees become empowered to take action on the social determinants of health in their future practice. The authors conducted a systematic review to better define the impact that educational programs centered on medical legal partnerships have on trainees’ knowledge, attitudes and future practice.

The authors sourced data from PubMed, Web of Science, Index to Legal Periodicals, LegalTrac, Google Scholar, Academic Search Complete, Business Source Complete, SocINDEX, SSRN, and Proquest Social Sciences. Selected studies included those centered on Medical Legal Partnerships in undergraduate or graduate medical education and that measured outcomes of the participating trainees. Two abstractors independently extracted information about the study population, setting, design, intervention and outcomes.

**Results:**

Six out of 483 studies met the inclusion criteria. One study highlighted four different MLPs, thus nine total MLP programs were included. Trainees included medical students as well as interns and residents from pediatrics, family medicine and internal medicine. Interventions ranged from didactic sessions, to advocacy projects, to hands-on community-based learning, to poverty simulation trainings. Benefits to trainees were wide in scope but all programs showed improvements in participants’ understanding, comfort, confidence, and/or abilities in identifying and intervening on the social determinants of health in their patients.

**Conclusion:**

As medical schools and residency programs are increasingly considering how to effectively teach trainees to understand and address the social determinants of health, the findings in this systematic review suggest that inclusion of Medical Legal Partnerships into training programs is an effective approach.

The social determinants of health (SDoH), such as substandard housing, psychosocial stress, and access & affordability of healthcare, are established risk factors that lead to worse health outcomes in disadvantaged populations [1]. An emphasis on addressing social determinants to improve the health of vulnerable populations is integral to many health initiatives around the world [2, 3]. Although healthcare providers also are increasingly recognizing that social determinants are important drivers of health in their patients and in their communities, the vast majority of providers do not routinely identify or address their patients’ social needs in clinical practice [4]. There are many barriers that explain this disconnect between best practice and reality, including providers’ lack of understanding of the importance of social issues, discomfort discussing these issues, constrained time and lack of education/knowledge of community resources available to help [5, 6]. To teach the SDoH effectively such that future doctors are empowered to take action on the SDoH in their future practice, educators will need to consider these barriers in their approach to SDoH training.

Fortunately, medical schools and residency programs are increasingly broadening the scope of SDoH educational approaches. Curricular interventions have included didactic sessions, service-learning, advocacy projects, experiential learning, and community partnered initiatives that collaboratively work to identify community needs and intervene through service or research [7, 8]. As connecting patients to community resources and non-physician experts on SDoH is an integral component of SDoH interventions [9], effective SDoH training programs need to engage in a team-based approach to train physicians to make an impact on health disparities.

A Medical Legal Partnership (MLP) is a team-based multidisciplinary intervention that incorporates co-located lawyers with SDoH expertise into a healthcare team [10]. Healthcare teams with MLPs can leverage legal advocacy to address challenging unmet social needs. As an example, an asthma patient who lives in a rented apartment with leaks, mold and an unresponsive landlord is unlikely to resolve their environmental triggers without legal advocacy.

Over two decades ago, the first MLP was formed by doctors at Boston Medical Center [11]. Since that time, the MLP model has been rapidly expanding, with hundreds of leading health organizations housing MLPs in diverse settings throughout the US [12], Canada and Australia [6]. As an increasing number of academic medical centers have incorporated MLPs into their operations. However, how to best incorporate MLP into medical education curricula designed to empower future providers to target SDoH is understudied. We conducted a systematic review to better define the value of MLPs multidisciplinary approach within medical educational curricula. In doing so, we also highlight innovative educational approaches taken by established MLPs. In reviewing this evidence base, we illustrate the impact of MLPs on the SDoH education of providers in training, reporting on future physicians’ understanding of, attitudes toward, and skills to address social determinants of health in their medical practice.

## Methods

We conducted a systematic review to better define the impact of curricular interventions within MLPs targeting the knowledge, attitudes and practice of MLP-engaged trainees with regard to the social determinants of health and health disparities. This was achieved by extracting information about study population, setting, design, intervention, and outcomes from studies that centered on MLPs in undergraduate and graduate medical education, as well as from those that measured outcomes of participating trainees.

The methodological quality of each study was assessed by two investigators (BR and KW) using the Mixed Methods Appraisal tool (MMAT), version 2018. The MMAT is a critical appraisal tool designed to evaluate the methodological quality of several types of empirical literature (i.e., qualitative, quantitative, and mixed methods studies) included in review studies. Agreement was reached on 76.67% of the appraisal items. Where scores differed, discrepancies were resolved through discussion. Quality score ranged from meeting none of the five criteria (0) to meeting all five criteria (5) [12].

### Literature search strategy

We designed our search strategy to be as inclusive as possible of published investigations of MLPs in the medical or legal literature. The following electronic databases were used in the search: PubMed, Web of Science, Index to Legal Periodicals, LegalTrac, Google Scholar, Academic Search Complete, Business Source Complete, SocINDEX, SSRN, and Proquest Social Sciences. Our search for articles was limited to those published in English. Given that MLPs are relatively new phenomena, we did not limit our search to a specific time period of publication. The search terms used were “medical legal partnership” or “medical legal partnerships.” In order to ensure we didn’t overlook any relevant studies, we also reviewed the references of all studies from our initial search and included any study not captured elsewhere. We did not include abstracts, unpublished data, or narrative accounts. Once we completed our initial search, we eliminated duplicate citations and imported the list of citations (211 unique references) into an EndNote X7 electronic database.

### Inclusion and exclusion criteria

We established a priori study eligibility criteria (Table 1). We sought to include studies that centered on Medical Legal Partnerships in undergraduate or graduate medical education. We included curricular interventions that measured outcomes of the participants engaged in the program. Measured outcomes centered on changes in knowledge, attitudes or practice by MLP-engaged medical students or medical residents.

**Table 1:** Inclusion and Exclusion Criteria

PICOTTSS CRITERIA	Inclusion	Exclusion
P: Population:	Undergraduate or Graduate medical education	
I: Intervention:	Medical Legal Partnerships programs integrated into educational curriculum in any capacity	
C: Comparator:	Any	
O: Outcomes:	**Knowledge or Attitudes** surrounding the importance of social determinants of health in patients / communities, as well the importance of legal aid / lawyers as team members on healthcare team **Practice** (including documentation) regarding any change in identifying or addressing unmet social needs in clinic patients	
T: Time over which to review literature:	Any	
T: Time allotted for outcomes to appear:	Any	
S: Study Designs Allowed:	Trials Cohort Studies Pre/Post single group	Case report or case series Systematic review Conference abstracts Review articles
S: Setting Allowed:	Any (E.g. hospital, clinic, community, hospice, VA, behavioral health)	
Publication Language	English	Any other

### Study selection

Two members of the team independently reviewed each title and abstract for eligibility (BR and KW). Reviewers resolved conflicts by discussion and a third reviewer (DH) adjudicated disagreements if needed. Three team members (DH, BR and KW) independently reviewed the full text of relevant articles and recorded the primary reason for article exclusion during the review process.

### Data extraction

Reviewers extracted data from each study meeting the inclusion criteria in a standardized process. Reviewers extracted data regarding the population and setting studied, the specifics of the intervention, the study design and study outcomes.

### Data synthesis and analysis

Upon systematically extracting the data, reviewers categorized the interventions and outcomes measured and used these categorizations to synthesize the data from the included studies.

## Results

**Fig. 1:**
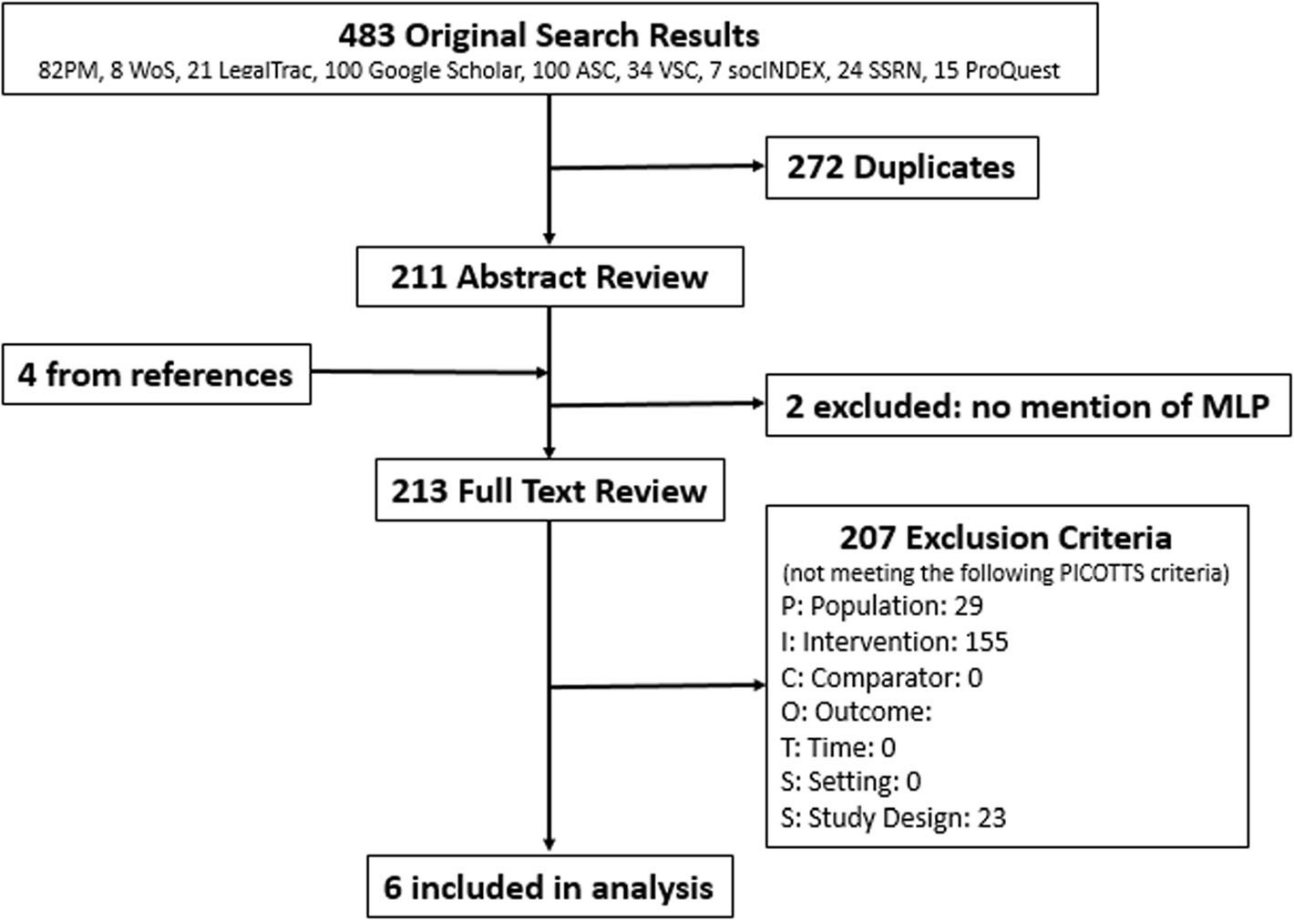
Article review flow diagram. Abbreviations: PM: PubMed, WoS: Web of Science, ILP: Index to Legal Periodicals, ASC: Academic Search Complete, BSC: Business Source Complete, SocINDEX, SSRN: Social Science Research Netwrk, and Proquest: Proquest Social Science

We identified 483 studies in our initial search and assessed 213 full-text articles for eligibility (Fig. 1). Using our inclusion and exclusion criteria, we excluded 207 articles. The vast majority of excluded studies (*N* = 155) were excluded due to not meeting the intervention criteria; in these studies, no specific intervention was analyzed. Twenty-nine articles did not meet the population criteria as they were not focused on medical education outcomes. Twenty three studies did not meet the study design criteria, as these studies were largely case reports/studies or reviews of related topics. Six studies were included in this systematic review. One of the included studies [13] highlighted four separate MLP programs that each met our inclusion criteria, thus we describe a total of nine MLP interventions.

### Characteristics of included studies

#### Population and settings

Sample sizes of interventions ranged from 19 to 143 (Table 2). Participants in the educational programs included medical students [17], internal medicine interns [13], and residents [13], family medicine interns [18], combined medicine-pediatrics residents [16], as well as pediatric interns [13, 15] and residents [14, 16]. Medical Legal Partnerships in all but one of the studies included a legal aid organization partnering with an academic medical center. In the MLP program that did not have a partnering legal aid organization, lawyers were integrated into the healthcare organization directly [18]. One MLP program also included a law school partnership [17].

**Table 2:** Characteristics of Medical Legal Partnership programs

Study	Population & Setting	Intervention	Study Design	Outcomes	Quality Rating^**a**^[12]
Klein, 2014 [14]	Pediatric residents (*N* = 47) Co-located MLP within a hospital based pediatric primary care center in Cincinnati Children’s Hospital partnered with the Legal Aid Society of Greater Cincinnati.	Delivery of a social determinants of health video curriculum by a multidisciplinary team (medical and legal experts). The curriculum featured clinical vignettes to highlight the importance of screening interventions targeting social determinants of health.	Non-randomized, controlled study Compared residents who received the video intervention to residents who did not (control) Surveys of patients and parents pre/post 6 month intervention Outcomes assessed: 1) Confidence in screening and intervening on behalf of SDH 2) Rates of referrals to MLP 3) Rates of referral for infant formula distribution for those who were food insecure	24 residents received the intervention, 23 residents received a standard curriculum (control) The intervention group was 1) More confident screening for housing, benefits and educational issues 2) More likely to screen for domestic violence and depression 3) Refer patients for formula distribution when food insecurity was found.	5
Klein, 2011 [15]	Pediatric interns (*N* = 38) Co-located MLP within a hospital based pediatric primary care center in Cincinnati Children’s Hospital partnered with the Legal Aid Society of Greater Cincinnati.	Delivery of a mandatory 2 week intern advocacy curriculum that incorporated a focus on the social determinants of health including shadowing social workers, guided tours of community organizations such as food banks and lectures taught by multidisciplinary experts (medical and legal) including topics such as the rationale behind MLPs, as well as technical and legal aspects of public benefits, housing and education.	Non-randomized, mixed methods study Compared interns who received the curricular intervention to the prior year interns who did not (control); Also assessed for pre/post change in the intervention group Outcomes assessed: 1) Knowledge surrounding benefits, housing and education 2) Attitude/Comfort assessing patients social needs 3) Documentation of social determinants in the EHR 4) Practice: referral rate to the MLP program	20 interns received the intervention, 18 interns received a standard curriculum (control). For both the pre/post comparison within the intervention group AND the intervention compared to control group, the intervention group: 1) More knowledgeable about benefits, housing and education 2) More comfortable discussing poverty issues; more likely to share information about relevant community resources; More likely to ask patients about social determinants including safe/stable housing and food insecurity The intervention group when compared to the control group was: 3) More likely to document issues related to benefits, housing and food insecurity 4) Had a trend toward increased referral rate to the onsite MLP (4% versus 2.9%, P = 0.13)	5
O’Toole, 2012 [16]	Pediatric and combined internal medicine/pediatrics residents (*N* = 40) Continuity clinics at 3 different sites that had varying levels of social service and legal supports within Cincinnati Children’s Hospital Center – ranging from a clinic with an onsite MLP with 2 lawyers, a paralegal as well as 3 full time social workers, to a clinic with no legal support and limited access to a social worker	None	Cross-sectional comparative survey of resident’s confidence and practice patterns identifying and addressing SDH Comparison between residents who had their continuity at different clinical sites stratified by the level of legal and social work support. Studied outcomes in residents at different clinical sites included differences in knowledge, attitudes and practice related to social determinants of health in their primary care practice.	When compared to residents who had their continuity clinic in a setting without co-located MLP, residents with access to co-located MLP: 1) Had increased confidence in their knowledge of benefits and food insecurity 2) Were more likely to ask patients about their housing, WIC (women infant and children program), public benefits and food insecurity 3) Spent a greater amount of time discussing social history with their patients	3
Pettignano, 2017 [17]	3rd year medical students (*N* = 100) MLP with Children’s Healthcare of Atlanta, Morehouse School of Medicine, the Atlanta Legal Aid Society and the Georgia State University College of Law	Delivery of a curriculum designed to educate students about MLPs and the ways in which they could collaborate with other professionals to address social determinants of health in their patients / clients	Pre/Post intervention study analyzing impact of 3 different cohorts of medical students over 3 years. Survey regarding the perceived benefits of an MLP and the importance of inter-professional practice as well as assesses subjects confidence regarding their ability to identify and address social determinants of health in their patients	After the intervention, students were more likely to: 1) Appreciate that social determinants such as access to public benefits, can impact the health of low-income patients 2) Screen patients for socioeconomic and legal issues related to income, education, family law, health insurance, public benefits, and supplemental security income / disability. 3) Refer patients to a legal resource when facing a patient with socioeconomic or environmental issues that may affect health	4
Cohen et al., 2010 [13]	Medical residents (*N* = 143) at three clinics participated in this study. LegalHealth, a division of New York Legal Assistance has weekly co-located MLP clinics at 16 different hospitals and clinics. Interns (*N* = 76) in the primary care internal medicine program participated in this study. MLP Boston has co-located clinics at Boston Medical Center and six community health centers. Pediatric interns (*N* = 19) participated in this study. Peninsula Family Advocacy Program is a collaboration between Lucile Packard Children’s Hospital at Stanford, Ravenswood Family Health Center in East Palo Alto, San Mateo Medical Center and the Legal Aid Society of San Mateo County. (This paper also describes the Legal Assistance to Medical Patients program partnering with Beth Israel Medical Center pediatric and medicine residency programs, but does not describe the impact of MLP educational initiatives)	Didactic sessions through grand rounds presentations such as “Bring advocacy to patients,” and one to one teaching sessions on topics such as income supports Multiple components including a poverty simulation session, and physician advocacy training which includes touring community resources and didactic sessions relevant to social determinants of health as well as clinical exposures with vulnerable populations Multiple components including an interdisciplinary course “medical-legal issues in children’s health,” and separate didactic sessions on topics such as “Immigrants and the health care system” and “your patient and the workplace,” as well as one on one sessions for a range of topics including legal status (e.g. immigration) and personal stability (e.g. advanced directives, guardianship)	Pre/Post intervention surveys surrounding knowledge, attitudes and practice in MLP Evaluations limited to surveys after the interventions and informal qualitative feedback Evaluations limited to surveys after the interventions and informal qualitative feedback	After completing the interventions, residents were: 1) More likely to believe it is the responsibility of the physician to help patients find free legal services when needed 2) Have increased knowledge regarding how to assist patients seeking public benefits 3) More likely to refer patients to legal services 4) More likely to assist patients with obtaining government benefits and obtaining safe housing After completing these programs: 1) 97% of participants reported they could screen for two unmet social needs 2) 74% strongly agreed and 21% somewhat agreed that they better understood poverty and the majority felt that “the experience has helped me better understand how poverty can affect health” Qualitative feedback included “I feel more encouraged in my ability as an MD to make changes” Interns attitudes towards legal screening for needs improved, as few providers reported concerns about making patients “nervous” with legal questions Qualitative feedback included “this course does a whole lot to empower students to effective action and advocacy” and “seeing how lawyers prioritize components of a patient case differently than physicians gave me a new perspective on how I might approach a patient.”	3
Pettit, 2019 [18]	Family medicine interns (*N* = 39) Tucson Family Advocacy Program MLP within the University of Arizona Department of Family and Community Medicine	A multidisciplinary (MLP director and medical director of primary care clinic) led advance care planning training program which included didactics and direct observations of residents conducting advance care planning discussions	Pre/Post intervention surveys related to residents comfort performing advanced care planning discussions MLP director ratings of residents during direct observations of residents conducting advanced care planning (scored according to ACGME milestone ratings during shadowed patient encounters).	Interns’ advanced care planning discussions with patients improved after receiving the intervention – During the first year of the program, residents were almost all rated as ACGME level 1 “beginner” or 2 “novice” and by the 3rd observed session, residents were all rated at least a ACGME level 3 “developing” which is the expected level for a 2nd or 3rd year resident. Residents also reported increased comfort leading advanced care planning discussions.	3

^a^ Quality score ranged from meeting none of five criteria (0) to meeting all criteria (5)

#### Interventions

Each MLP program in this analysis had multifaceted and multidisciplinary curricular interventions centered on the social determinants of health and health disparities. The term “intervention” is used to describe these programs in the research setting, but in a plain language setting, they might simply be called educational programs, initiatives, or SDoH curricula. Interventions were varied and ranged from didactic trainings [13–15, 17, 18], to one-on-one educational sessions in the context of patient care [13, 15, 18], to longitudinal experiences [16]. Some interventions were university/medical center-based entirely [16, 18], occurring in the same locations trainees already work, while others included community based activities such as a poverty simulation and tours of community-based organizations that address social needs such as food banks or homeless shelter s[13, 14]..

#### Study designs

Two studies were non—randomized controlled studies that compared residents who received a MLP intervention to residents who did not receive the MLP intervention (e.g. control group) [14, 15]. Three studies were pre/post intervention measuring the impact of the intervention on the same group of residents over time [13, 17, 18]. One study was a cross sectional study that compared residents at different clinical sites with varying levels of social work and legal aid support, ranging from having a co-located MLP with multiple social workers and lawyers on site, to a clinical site without co-located MLP services (but remote access) [16]. Two programs described the results of surveys completed by residents who were evaluating their experience within a MLP curricular intervention [13].

The evaluations for each MLP study in this analysis utilized surveys to assess residents’ and students’ knowledge, attitudes and practice of identifying and intervening on social determinants of health. One study incorporated direct observations of residents’ patient care interactions to gauge the effectiveness of the skills learned in MLP didactic sessions [18].

#### Outcomes

Each study used different measures of determining the effectiveness of their interventions. However, each study demonstrated improvements in some aspects of knowledge, attitudes or practice regarding participants’ understanding, comfort, confidence and abilities in identifying and intervening on the social determinants of health in their patients. Because of this diversity in methods and measures, we will describe each study’s findings individually and then synthesize common themes among them.

In one non-randomized controlled study conducted by Klein et al., pediatric residents received intervention via delivery of a video curriculum by a multidisciplinary team of medical and legal experts that featured clinical vignettes highlighting the importance of screening interventions that target SDoH [14]. Residents completed self-assessments on screening competence and resource knowledge, and patients and families were also surveyed about their visits [14]. Compared to residents who did not receive the intervention, residents in the intervention group were more confident in screening for housing, benefits, and educational issues (each *P* ≤ .05); more likely to screen for domestic violence (odds ratio 2.16, 95% confidence interval 1.01–4.63) and depression (odds ratio 2.63, 95% confidence interval 1.15–5.99); and more likely to refer patients for formula distribution when food insecurity was found (*P* = .02, 14]. This study is limited by the fact that participants were not blinded and outcomes were measured with self-assessments which could have contributed to bias, however these results demonstrated that SDoH video curricula improved self-assessed screening competence and formula distribution.

In another study by Klein et al., pediatric interns attended a two-week advocacy curriculum that incorporated a focus on SDoH. This curriculum included shadowing social workers, guided tours of community organizations, and lectures given by a multidisciplinary team of medical and legal experts on MLP and the technical and legal aspects of public benefits, housing, and education [15]. Anonymous online surveys were collected at the end of the intern year and results were compared to a control group of pre-intervention self-assessments and surveys of prior year interns who did not receive the curriculum [15]. Compared to the control group, intervention interns felt more knowledgeable about benefits (72% vs 52%), housing (48% vs 21%), and education (52% vs 33%, *P* < .001 for all); more comfortable discussing poverty issues (100% vs 71%, *P* < .01); and were more likely to document each issue (benefits 98% vs 60%, housing 93% vs 57%, food 74% vs 56%; P < .001 for all) [12]. The intervention group also had a trend toward increased MLP referral rate though the difference was not statistically significant (4% versus 2.9%, *P* = 0.13, 13]. The strength of this study’s results are limited by the use of self-assessments, but the results suggest educational intervention can increase comfort and knowledge about SDoH and community resources among interns.

O’Toole et al. conducted a cross-sectional comparative survey of the confidence and practice patterns of pediatric and combined internal medicine and pediatric residents in identifying and addressing SDoH [16]. Residents were located at one of three continuity clinics that had varying levels of social service and legal support by an onsite MLP [16]. Using resident surveys and direct observations of residents during visits, this study found that residents who had access to more social and legal services had increased confidence in their knowledge about resources, were more likely to ask patients about their housing, public benefits, and food security, and spent a greater time discussing social history with their patients [16]. Limitations of this study include differences in additional training experiences that Med-Ped residents may have had on their internal medicine rotations, a limited number of observations in the given study period, and failure of faculty observers to be blinded to the clinic location during observations [16].

In the study performed by Pettignano and colleagues, a four-session didactic interprofessional curriculum was implemented by medical educators and MLP faculty for third year medical students and co-attended by law students. The curriculum involved 8h total over the academic year, and included large group presentations by doctors and lawyers, small group discussions, mixed-discipline discussions, and case studies, all pertaining to a wide array of medicolegal issues that impact patients [17]. Pre and post intervention surveys were administered to 222 medical students over the 4 year study period, with a 46 and 45% response rate for pre and post surveys, respectively. After the curriculum, medical students self-reported an increased likelihood to screen and assist patients for socioeconomic and legal issues in the areas of income, education, family law, health insurance, public benefits, and social security disability insurance (*p* < 0.05). The greatest change in attitude post-intervention (increase in greater than 25%) was referring patients to a legal resource when aware that their patients were experiencing socioeconomic, environmental, or legal issues that were affecting their health [17]. This indicates the curricular intervention for medical students increased the likelihood patients facing these issues would receive the legal assistance they needed. This study was limited by relative low survey response rates. The results of this study may also have been impacted by the study location (Morehouse Medical College) which has a stated mission of assisting low-income populations.

Cohen and colleagues detailed the impact of educational efforts by four separate MLPs and the results of preliminary pilot studies there [13]. All four programs incorporated didactic sessions and direct one-on-one teaching around cases on a wide variety of MLP-related topics such as housing, workplace, immigrants and health care, and legal decision making. Two of the programs had block courses or available rotations at the MLP for their residents. In one MLP training curriculum with didactic and one-on-one teaching, residents completed pre and post surveys (33% response rate) which showed, after completing the MLP educational initiative, more residents believed it was the responsibility of physicians to help patients find free legal services when needed (21 to 52%); were more likely to refer patients to legal aid (15 to 54%), help patients obtain government benefits (45 to 54%), and obtain suitable housing (24 to 37%); and finally had more knowledge about how to assist patients seeking public benefits (42 to 62%) [13].

Another well-established MLP used didactic teaching, one-on-one learning, a required 4 week rotation, and community work to teach residents about SDoH. The required rotation included clinical experiences with vulnerable populations, such as those experiencing homelessness, lectures about advocacy and the legislative process, tours of community facilities such as homeless shelters and housing court, as well as a project to address a socially caused health disparity. Qualitative evaluations revealed that residents in this MLP curricular initiative often felt more empowered to advocate for the social and legal needs of their patients after program participation [13]. Thirty-five interns also participated in a 2 h poverty simulation exercise, and 95% agreed the experience helped them understand poverty, with 100% agreeing it helped them understand how poverty can affect health [13]. This MLP also held a 3 h advocacy training session for physicians and allied health providers. On post-session surveys, (84–100% response rate), 83–89% of providers stated that they would make changes to their practice based on what they had learned [13].

One MLP described by Cohen and colleagues found that interns (response rate ~ 20%), had improved attitudes towards screening for legal needs, with fewer reporting concerns about making patients nervous (38 to 21%) after didactic training developed by MLP faculty on social and legal issues [13]. This MLP program also developed an interdisciplinary course for law and medical students about pediatric medical-legal issues, which involves learning about SDoH and advocacy skills, as well case discussions and policy work. Qualitative feedback demonstrated improved collaboration with lawyers to assist patients and increased empowerment to advocate for patients [13]. An MLP associated with both medical and pediatric residency programs, using didactics and one-on-one teaching, did not have follow-up survey data available to assess the impact of its educational initiatives. The four programs described in the study by Cohen and colleagues were generally limited by lack of control groups and the pilot study-nature of the interventions.

In the study by Pettit and colleagues, a lawyer and a physician from an academic MLP developed an educational program intended to train first year residents to lead advanced care planning (ACP) discussions, a task which involves medical-legal elements. The initial program was 1 h long and included various didactic materials including handouts, multimedia presentations, and example videos. After feedback about the need for further training, more specific didactic materials were included, and direct observations of residents leading ACP discussions were performed and scored [18]. After the initial training, residents showed only small improvements in self-reported knowledge about and confidence with ACP discussions on comparison of pre- and post-session surveys [18]. However, after additional instruction and either two or three observed encounters coupled with verbal feedback, evaluation of residents’ ACP discussions by observers, using an evaluation tool based on the ACGME milestone system, generally improved, although statistical analysis was not performed due to small sample size (number of encounters = 39, number of residents = 15). Residents’ self-evaluation after intervention suggested they felt fairly or very confident when discussing ACP topics. This study suggests MLP may be useful in teaching residents about advanced care planning discussions with patients. However, it was limited by a small sample size, lack of control group, and lack of blinding procedure for evaluation. Thus these findings are preliminary and require further investigation.

Overall, evidence is strong that MLP-led educational initiatives can make a positive impact on the awareness of providers towards the impact of social and legal issues on health, and on the likelihood that providers will provide more robust resources to assist patients with these issues, through direct assistance or referrals [13–17]. This impact was achieved through various types of educational activities in these studies, including didactic learning [13–15, 17] and active learning such as case discussions [13, 17], experiences in community settings [13, 15, 16], one-on-one teaching [13], and having a MLP co-located in clinics [16]. This impact was largely measured by self-assessments surveys [13–18] and a small number of clinical observations [16, 18].

The evidence is not definitive about which MLP-based educational initiatives and interventions are most effective. Each program had a different way of training providers and/or students to most effectively address SDoH, and no true comparative studies were done. One study did show that working in a clinic with an MLP present versus a clinic with no on-site MLP was more effective at increasing providers’ confidence in and likelihood of addressing SDoH [16]. There were various intensities of intervention, with some initiatives requiring 4 week rotations, and others delivering 2–3 h of didactic teaching. Objective assessment of the development of skills with MLP-based educational initiatives was performed in two studies, with modest improvements shown in performance related to addressing SDoH [16] and discussing advance care planning [18]. The available evidence did not strongly suggest a mechanism for MLP impacts on SDoH education, as studies largely reported on attitudes, knowledge, and practices pertaining to SDoH post-MLP education intervention rather than delving into deeper analysis of the education provided. In this aspect, the evidence surrounding MLP-based education is in its nascent form.

## Discussion

To our knowledge this is the first systematic review of undergraduate and graduate medical education curricular interventions that center on Medical Legal Partnerships targeting social determinants of health. In this review of eight MLP programs, all interventions were effective at improving participants’ knowledge, attitudes and/or practice regarding issues related to the SDoH and health disparities. These improvements were wide in scope and included benefits in participants’ understanding, comfort, confidence, and abilities in identifying and intervening on the social determinants of health in their patients. Participants from multiple disciplines including family medicine, pediatrics and internal medicine residencies, as well as undergraduate medical students each demonstrated benefit from the MLP-centered interventions. Students and residents engaged with MLPs through varied activities built into an SDoH curriculum including didactics, advocacy training, and interactive programs such as a poverty simulation. Multiple MLP programs highlighted in this review brought learners out of the ivory towers of academia and into the community to learn about, and collaborate with, community based resources. In doing so, MLPs help bring learners closer to the context where risks emerge, and foster collaboration with key change agents [19].

Even with a targeted curriculum, effective training on SDoH that impacts health disparities is no easy task; this study highlights the important role that MLPs may provide in addressing this difficult topic in medical education. As teaching the SDoH to impact health disparities is relatively new in medical education, there is a limited evidence base to guide and assess effective curricular development. Thus, it is important to consider teaching the SDoH through MLP in the context of known effective medical education approaches that target other health topics with the goals of better health outcomes.

First, understanding causal pathways is critical for learners to comprehend, retain, apply and advance most topics in medicine [20]. Causal pathways in the SDoH and health disparities are no exception. An understanding of the systems, laws and policies that are in part responsible for ongoing disparities in environmental, behavioral or medical determinants is important to integrate into medical education curriculums. As highlighted in each of the studies within this review, lawyers focused on caring for vulnerable populations through legal advocacy are well-equipped to provide this education within the context of a MLP [14, 15, 17, 18, 21, 22].

Second, to impact health disparities through education, it is critical to teach students actionable and practical skills to help address SDoH [7]. In traditional medical education, learners gain these skills by practicing medicine in closely supervised environments alongside of supervisory clinicians (e.g. clinical rotations in medical school or residency training). Similarly, MLPs provide a unique opportunity for which learners can foster advocacy skills by working alongside a lawyer with experience and expertise in advocacy. In many of the above-described MLP programs, learners work one-on-one with lawyers, and gain confidence in ameliorating unmet social needs in their patients through real patient encounters.

Third, simulation training is a widely established tool in medical education that can help learners experience a virtual reality through which they gain situational awareness, enhance communication skills and learn through a cycle of feedback and debriefing [23]. Simulation trainings, such as the poverty simulator which was incorporated in the Boston Medical Center MLP program described in this review [13], can be an effective experiential learning tool within SDoH training [24]. For learners who might not have previously experienced poverty, a simulator, which includes an interactive immersion experience, can sensitize participants to the ways in which their patients are constrained and shaped by economic and political forces [25]. An improved understanding of these structural challenges is an important aspect of training physicians to become advocates for the needs of their most vulnerable patients [25, 26].

As educators consider how they will incorporate SDoH into their curriculums, the opportunity to partner with MLPs is timely as MLPs are increasingly becoming an important component of multidisciplinary clinical care teams around the country. Over 400 health care organizations have developed MLPs in 48 states in the US and most have formed in the past decade [27]. Many MLPs, including all of the presented programs in this review, operate in partnership or within an academic medical center which facilitates the engagement of undergraduate and graduate medical trainees into multidisciplinary programs.

This systematic review has several limitations. First, we included all MLP studies that focused on medical education outcomes. These studies are heterogeneous in their intervention, design and evaluation. This heterogeneity limited our capacity to conduct a quantitative meta-analysis. Second, while we employed a comprehensive search strategy of various databases with the help of an experienced medical librarian, it is possible that we did not capture all relevant articles or programs. Our literature search included a single term, “medical legal partnership,” and therefore similar arrangements, especially in international settings, labeled as “health justice partnerships” or “advocacy health alliances,” for example, would not be included in our analysis. Third, there were no studies that compared the MLP intervention directly with other forms of social determinants of health training. However, the qualitative study results presented above highlight the importance of the multi-disciplinary perspective in SDoH curricula: Having a legal expert on the team positively impacted measured outcomes including participants’ perceived ability make a difference as a physician. Lastly, most MLPs around the country have been formed in the last decade and research into the impact of MLP on patient care outcomes is nascent. Several of the included studies analyzed the impact on student or resident behavior, such as demonstrating improvement in documentation surrounding SDoH in the electronic record [15] or increased likelihood of referrals to community organizations including legal aid [13]. However, more rigorous, quantitative analysis exploring the impact of MLP educational programs on participants’ future practice is warranted. Studying the impact of MLP programs on patient outcomes outside of a medical educational program was beyond the scope of this review.

## Conclusion

As medical schools and residency programs around the US are increasingly considering how to include educational programs targeting the social determinants, the findings in this systematic review suggest that Medical Legal Partnership centered interventions should be considered in both undergraduate and graduate curricula. As medical legal partnerships continue to grow in size, scope and location around the country, the opportunity to engage MLPs into medical educational curriculum will expand. More studies evaluating the impact of these educational programs centered on MLP are needed, including studies that document changes in student and resident practice.

## Data Availability

Not applicable.

## References

[1] Silversein M, Hsu HE, Bell A. Addressing the social determinants to improve population health: the balance between clinical care and public health. JAMA. 2019;322(24):2379–80. 10.1001/jama.2019.18055 31790542

[2] Secretary’s Advisory Committee on Health Promotion and Disease Prevention Objectives for 2020 (2010). Healthy people 2020: an opportunity to address the societal determinants of health in the United States.

[3] Marmot M, et al. Closing the gap in a generation: health equity through action on the social determinants of health. Lancet. 2008;372(9650):1661–9. 10.1016/S0140-6736(08)61690-6 18994664

[4] Fraze TK (2019). Prevalence of screening for food insecurity, housing instability, utility needs, transportation needs, and interpersonal violence by US physician practices and hospitals. JAMA Netw Open.

[5] Garg A (2009). Screening for basic social needs at a medical home for low-income children. Clin Pediatr (Phila).

[6] Schubert C, et al. Teaching Advocacy to Physicians in Multicultural Settings. Open Medical Educ J. 2009;2:36–43.

[7] Sharma M, Pinto AD, Kumagai AK (2018). Teaching the social determinants of health: a path to equity or a road to nowhere?. Acad Med.

[8] Seifer SD (1998). Service-learning: community-campus partnerships for health professions education. Acad Med.

[9] Davidson KW, McGinn T. Screening for social determinants of health: the known and unknown. JAMA. 2019;322(11):1037–8. 10.1001/jama.2019.10915 31465095

[10] Regenstein M (2018). Addressing social determinants of health through medical-legal partnerships. Health Aff (Millwood).

[11] Goldberg C. Boston Medical Center turns to lawyers for a cure. The New York Times. 2001. Available from: https://www.nytimes.com/2001/05/16/us/boston-medical-center-turns-to-lawyers-for-a-cure.html.

[12] Hong QN, P.P., Fabregues S, Bartlett G, Boardman F, Cargo M, Dagenais P, Gagnon M-P, Griffiths F, Nicolau B, O'Cathain A, Rousseau M-C, Vedel I. , *Mixe Methods Appraisal Tool (MMAT), Version* 2018*.* Registration of copyright (#1148552)*,* Canadian Intellectual Property Office*,* Industry Canada*:* http://mixedmethodsappraisaltoolpublic.pbworks.com/w/file/fetch/127916259/MMAT_2018_criteria%20manual_2018%202008%202001_ENG.pdf (Accessed 30 Mar 2021).

[13] Cohen E (2010). Medical-legal partnership: collaborating with lawyers to identify and address health disparities. JGIM: J Gen Intern Med.

[14] Klein MD (2014). Can a video curriculum on the social determinants of health affect residents' practice and families' perceptions of care?. Acad Pediatr.

[15] Klein MD (2011). Training in social determinants of health in primary care: does it change resident behavior?. Acad Pediatr.

[16] O’Toole JK (2012). Resident confidence addressing social history: is it influenced by availability of social and legal resources?. Clin Pediatr.

[17] Pettignano R (2017). Interprofessional medical-legal education of medical students: assessing the benefits for addressing social determinants of health. Acad Med.

[18] Pettit JM (2019). Medical-legal partnerships to enhance residency training in advance care planning. Fam Med.

[19] Klein M, Beck AF (2018). Social determinants of health education: a call to action. Acad Med.

[20] Sibbald M, Neville A (2016). A hundred years of basic science in medical education. Perspect Med Educ.

[21] O'Toole JK (2012). Resident confidence addressing social history: is it influenced by availability of social and legal resources?. Clin Pediatr (Phila).

[22] Cohen E (2010). Medical-legal partnership: collaborating with lawyers to identify and address health disparities. J Gen Intern Med.

[23] Akaike M (2012). Simulation-based medical education in clinical skills laboratory. J Med Investig.

[24] Hsieh DT, Coates WC (2018). Poverty simulation: an experiential learning tool for teaching social determinants of health. AEM Educ Train.

[25] Metzl JM, Hansen H (2014). Structural competency: theorizing a new medical engagement with stigma and inequality. Soc Sci Med.

[26] http://www.povertysimulation.net/*.*

[27] The National Center for Medical Legal Partnerships: https://medical-legalpartnership.org/, accessed 19 Feb 2020*.*

